# Patient Involvement in the Rehabilitation Process Is Associated with Improvement in Function and Goal Attainment: Results from an Explorative Longitudinal Study

**DOI:** 10.3390/jcm13020320

**Published:** 2024-01-06

**Authors:** Joachim Støren Sagen, Ingvild Kjeken, Andreas Habberstad, Anita Dyb Linge, Ann Elisabeth Simonsen, Anne Dorte Lyken, Eirik Lind Irgens, Heidi Framstad, Peter Solvoll Lyby, Mari Klokkerud, Hanne Dagfinrud, Rikke Helene Moe

**Affiliations:** 1 Faculty of Health Sciences, Oslo Metropolitan University, St. Olavs plass 4, 0130 Oslo, Norway; mariklok@oslomet.no; 2 Diakonhjemmet Hospital, Center for Treatment of Rheumatic and Musculoskeletal Diseases (REMEDY), Norwegian National Advisory Unit on Rehabilitation in Rheumatology (NKRR), Diakonveien 12, 0370 Oslo, Norway; ingvild.kjeken@diakonsyk.no (I.K.); h.s.dagfinrud@medisin.uio.no (H.D.); rikmoe@gmail.com (R.H.M.); 3 The Norwegian Federation of Organizations of Disabled People, Mariboesgate 13, 0183 Oslo, Norway; andreas.habberstad@ffo.no; 4 Muritunet Rehabilitation Center, Grandegata 58, 6210 Sylte, Norway; anita.dyb.linge@muritunet.no; 5 Røysumtunet Rehabilitation Center, Røysumlinna 41, 2770 Jaren, Norway; aesimon77@gmail.com (A.E.S.); hf@roysumtunet.no (H.F.); 6 Sørlandet Rehabilitation Center, Ola Garsons vei 1, 4596 Eiken, Norway; anne.dorte.lyken@sorrehab.no; 7 Helsepartner Rehabilitation Center, Follumsvei 1, 9510 Alta, Norway; eirik.irgens@hpnn.no; 8 Catosenteret Rehabilitation Center, Kvartsveien 2, 1555 Store Brevik, Norway; peter.lyby@catosenteret.no

**Keywords:** patient engagement, goal-setting, health care services, rehabilitation, goal attainment, shared decision making, RMDs

## Abstract

The objective was to explore the associations between patient involvement in the rehabilitation process and improvements in function and goal attainment in the first year after rehabilitation. The longitudinal multicenter study RehabNytte provided data from participants who had been referred to rehabilitation (*n* = 2113). Quality indicator (QI) pass rates (% yes) were used to assess patient involvement in the rehabilitation process. The Patient-Specific Functional Scale (PSFS) (10 = best possible) was used to assess function. The outcome QI on goal achievement (response options of yes/no) was used to assess goal attainment. Logistic regression and paired sample t-tests were used to examine associations and mean changes in function from rehabilitation admission up to 3, 6, and 12 months. Most participants (95%) were involved in goal-setting, which was positively associated with younger age (OR 0.97, 95% CI 0.95–0.99) and female sex (OR 1.87, 95% CI 1.15–3.02). Function improved over the follow-up period, with greater improvements in the active goal-setting group. Being involved in goal planning almost tripled the odds of goal attainment (OR 2.78, 95% CI 1.60–4.83) and involvement in the rehabilitation plan almost doubled it (OR 1.99, 95% CI 1.41–2.81). Most participants were involved in rehabilitation goal-setting/planning and being involved was associated with beneficial functional outcomes and greater goal attainment.

## 1. Introduction

A participatory healthcare approach is considered important for enhancing the quality of healthcare services [1]. Patient engagement (PE) is part of this approach. PE can be described as facilitating processes that strengthen the capacity for patients to be actively involved in decisions about their care [2] and include active involvement at different levels of healthcare [3]. The micro-level is suggested to include direct care, involving person-to-person collaboration, for example, between patients and healthcare professionals. At the micro-level, patients are involved in their own health decisions such as goal-setting processes. The meso-level refers to the involvement of patients as representatives in the development and delivery of healthcare services and systems, such as structural healthcare attributes, processual activities, tasks, and behaviors of the involved stakeholders. PE at the meso-level is often organized into patient advisory boards, councils, or forums [4,5]. As co-creators of political incentives at the macro-level, patient representatives often represent patient organizations or patient advocacy groups to highlight the groups’ special interests [5].

Even if patient involvement is considered a criterion for the quality of healthcare services [6], meaningful involvement faces several barriers [7]. Patients’ health literacy and their desire to be actively involved in their health vary and healthcare professionals have reported a lack of knowledge regarding the implementation of PE [8]. Additionally, healthcare systems often lack the necessary structures to promote PE effectively [9,10,11].

In rehabilitation processes, active involvement in goal-setting and the development of goal-driven rehabilitation plans are key components of PE at the micro-level [8,12]. The process of short- and long-term goal-setting can be patient-directed, mutually agreed upon, or staff-driven [13].

In Norway, patients have a legal right to be involved at every level of healthcare [14]. The basic assumption is that facilitating structures at the macro- and meso-levels enhances patient involvement at the micro-level, which again may be associated with improvements in functioning, goal attainment, and quality of life [15,16,17]. However, there is a lack of evidence supporting the associations between meso- and micro-level PE and additional research is needed [11]. Hence, the aim of this study was to explore the possible associations between demographic characteristics and patient involvement in setting rehabilitation goals and between patient-reported involvement in the rehabilitation process (reflecting the implementation of meso-level structures) and the changes in function and goal attainment in the first year after rehabilitation (micro-level).

## 2. Materials and Methods

### 2.1. Study Design, Participants, Setting, and Data Collection

**Figure 1:**
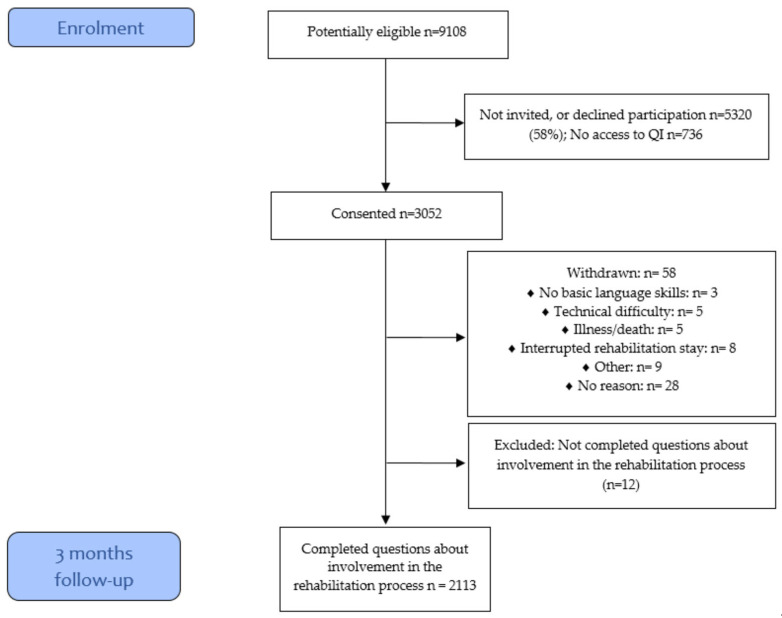
Participant flowchart.

The current study is part of the RehabNytte study, which is a large prospective longitudinal multicenter study with a 12-month follow-up period. In total, more than 3000 participants ≥18 years old with different diagnoses who received rehabilitation at 17 rehabilitation centers from all Norwegian health regions were included between January 2019 and March 2020. Patients were invited to participate during their first encounter with a healthcare professional at the point of admission to rehabilitation and were given oral and written information before providing informed consent. The centers offer inpatient and outpatient rehabilitation, target various diagnoses, and also have different rehabilitation specialties, contents, facilities, and resources. A flowchart outlining the data collection process in the current study is shown in Figure 1. The data were collected through a web-based portal that required authentication in accordance with GDPR data security level four. Collection was performed at admission (baseline) and at discharge and at 3, 6, and 12 months after admission to rehabilitation.

### 2.2. Variables

The demographic variables included sex, age, living with a partner (yes/no), duration of pain ≥3 months (yes/no), and work status (full-time work/part-time work/not in paid work). Education was registered as education ≥12 years (yes/no). The variable diagnosis was collapsed from twenty-six categories into four main categories comprising the three most common diagnoses as follows: rheumatic and musculoskeletal diseases (RMDs), lifestyle-related diseases (primarily overweight), neurological diseases, and “other”. The “other” category included smaller diagnostic groups, such as those with sensory disabilities and multiple types of trauma. Age was categorized into 17–39 years, 40–49 years, 50–59 years, 60–69 years, and 70–>80 years.

To measure PE, we used a quality indicator (QI) set developed for the rehabilitation of people with RMDs [18] and tested for responsiveness with good results [19]. The QI set comprises 19 items related to structure, 11 related to process, and 3 related to outcomes of the rehabilitation process. The QIs reflect the quality of care at a meso-level [20] where structural indicators are reported by leaders at the rehabilitation centers (meso-level), whereas process indicators are reported by patients responding with a yes or no to each item, thereby reflecting patients’ experiences of the implementation of structures for the PE process at the meso-level. In the present study, the following five QIs were included: “Were you actively involved in setting specific goals for the rehabilitation?”, “Were you actively involved in preparing a specific written plan for the rehabilitation period?”, “Did you participate in at least two meetings with the team (or a health professional representing the team) during which your goal(s) and goal attainment thus far were discussed?”, “Were you asked if you wanted attendance in any of the meetings for your next of kin?”, and “Were you asked if you wanted attendance in any of the meetings for professionals you would be related to after the rehabilitation period?” (e.g., external healthcare personnel, a general practitioner, a social worker or someone from the workplace to participate in their rehabilitation meetings). We calculated the pass rates for each indicator as the total number of patients who answered “yes” for this particular indicator divided by the total number of responses for the same indicator. The pass rate scores were thereafter normalized to 100 to report the values as a percentage (0–100%, with 100% being the best score). The first process QI was additionally used as a measure of involvement in goalsetting, whereas the outcome QI of “as a result of the rehab period, have you achieved one or several goals that are important to you”, was used as a measure of individual goal attainment (micro-level) [18].

Functioning was measured with the Patient Specific Functioning Scale (PSFS), which was randomly allocated to half of the participants as part of the larger RehabNytte study. In the PSFS, patients list up to five important activities that they currently find difficult to perform due to their health condition. The experienced performance of each activity is scored on an 11-point scale (0–10), with 0 indicating “unable to perform” and 10 indicating “no problem at all”. The mean activity scores were thereafter calculated [21].

### 2.3. Statistics

Descriptive statistics are presented as the frequency and percentage or mean/median, as applicable. Groups were compared using t- tests or chi-squared tests. Paired sample t-tests were used to explore changes in function from baseline to 3, 6, and 12 months after rehabilitation admission. Logistic regression analyses were used to examine associations between the dependent variable of “active involvement in goal-setting” and patient characteristics (independent variables) as well as associations between the dependent outcome variable of “goal attainment” and patient characteristics and patient involvement in the rehabilitation process (independent variables). The results are presented as the means with standard deviations or as odds ratios with 95% confidence intervals. Both regression analyses and paired sample t-tests were two-sided, and the alpha level was set to *p* ≤ 0.05. All the statistical analyses were performed using IBM SPSS Statistics version 28, Armonk, NY, USA [22].

### 2.4. Ethics

The study was carried out following the principles outlined in the Helsinki Declaration and was designed according to the Personal Data Act and the General Data Protection Regulation (GDPR). The study was recommended by the data protection officer at Diakonhjemmet Hospital (DS-00040), dated 17 October 2018, and registered at ClinicalTrials.gov (NCT03764982). It was considered by the regional ethical committee to not require approval since the overarching goal was to evaluate the delivery of rehabilitation services (2018/1645/REK South-East A). The study participants were covered by the Norwegian System of Patient Injury Compensation while they were staying at the rehabilitation institution. The electronic data collection system used was provided by CheckWare and inclusion protocols were stored in lockable cabinets in the local project coordinator offices during the project period. When the data collection was completed, de-identified data were transferred to the research server at Diakonhjemmet Hospital and the code list was stored on a locked, encrypted USB flash drive. All identifiable data files will be anonymized within five years after completion, and the final report will be sent to the data protection office no later than 2027.

### 2.5. Patient Research Partner Involvement

Two patient research partners were engaged in all phases of the research project, including ensuring the use of inclusive language in the final manuscript and developing plain language summaries in Norwegian and English. They as well as the rehabilitation centers that participated in the study will also provide valuable support in sharing the results and putting the findings into practice. All patient research partners were included as authors following the Vancouver Declaration.

## 3. Results

A total of 2113 participants reported the QI score for involvement in goal-setting and 2100 reported the QI score for goal attainment. The mean age was 53 years (SD = 13.5) and the majority of participants were women (70%). The vast majority (92%) reported long pain durations (≥3 months) before rehabilitation, approximately half of the participants had ≥12 years of education, and half of the participants were working before rehabilitation admission. The largest diagnostic groups were RMDs (53%), lifestyle diseases (13%), and neurological injuries and diseases (11%) (Table 1).

### 3.1. Patient Involvement in the Rehabilitation Process

A total of 95% of participants reported being involved in setting rehabilitation goals, 79% reported being involved in preparing their rehabilitation plan, and 84% reported having participated in at least two rehabilitation meetings. However, 23% were asked if they wanted attendance at any of the meetings for their next of kin and 19% were asked if they wanted attendance of professionals whom they would relate to after the rehabilitation period. Compared with participants in the highest age group (89%), a greater percentage of participants in the younger age group were involved (95–97%) in goal-setting (Table 2).

### 3.2. Associations between Patient Characteristics and Involvement in Goal-Setting

Ninety-five percent reported being actively involved in setting rehabilitation goals. There was a greater likelihood of involvement among women than men (odds ratio [OR] 1.87, 95% CI 1.15–3.02) and among participants of younger ages (OR 0.97, 95% CI 0.95–0.99) (Table 3).

### 3.3. Associations between Involvement in Goal-Setting and Improvements in Functioning

**Figure 2:**
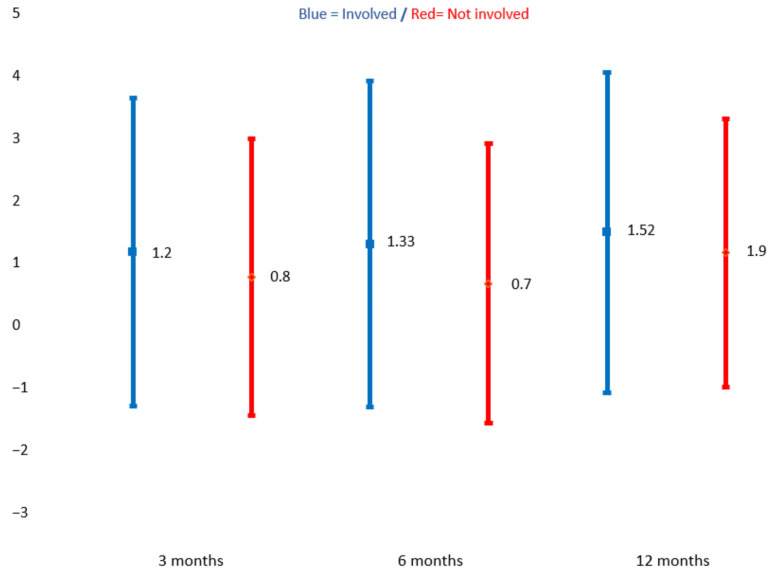
Mean change for those involved and not involved in goal-setting 3, 6, and 12 months after baseline. Patient-specific functional scale (scored 0–10, with 0 being the worst possible function). □ = involved in goal-setting, ◊ = not involved in goal-setting. Paired sample *t*–test.

A total of 779 participants completed the PSFS at baseline of which 778 were included in the analyses of changes after 3 months, 591 after 6 months, and 584 after 12 months. In general, there was a small mean improvement over the one-year follow-up period among participants. However, compared with participants reporting no involvement in goal-setting, those who were actively involved had greater improvement at all time points, with a significant improvement from baseline to 12 months [19] (Figure 2).

### 3.4. Associations between Patient Characteristics, Involvement in the Rehabilitation Process, and Goal Attainment

There were positive associations between goal attainment and higher age (OR 1.03, 95% CI 1.02–1.04) and having a lifestyle disease (OR 4.93, 95% CI 2.35–10.35). Compared with having less than 12 years of education, having higher education was negatively associated with goal attainment (OR 0.71, 95% CI 0.53–0.95), whereas having less than three months of continuous pain before rehabilitation admission was positively associated with goal attainment (OR 2.17, 95% CI 1.17–4.00). When examining the associations between involvement in the rehabilitation process and goal attainment, being involved in formulating goals (OR 2.79, 95% CI 1.60–4.87) and preparing the rehabilitation plan (OR 2.04, 95% CI 1.44–2.87) were positively associated with goal attainment (Table 4).

## 4. Discussion

The main goals of this study were to explore the associations between patient involvement in the rehabilitation process and improvements in function and goal attainment in the first year after rehabilitation. The results showed that most participants were involved in goal-setting, developing a rehabilitation plan, and in multidisciplinary team meetings, which are important elements of the rehabilitation process. In contrast, only about one in five participants were asked if they wanted their next of kin or health professionals outside the rehabilitation institution to attend any meetings; these elements may be important for ensuring the follow-up and implementation of lifestyle changes and self-management strategies initiated during the time spent by the patients in rehabilitation [23,24,25,26,27]. These results align with those found in prior studies [15,18,28], suggesting that while coordination across services is recognized as a crucial aspect of a high-quality rehabilitation process, it appears to be one of the weakest elements within the rehabilitation trajectory.

Engaging the relevant stakeholders as part of the rehabilitation process in secondary care has been shown to facilitate rehabilitation follow-up in primary care [29]. Additionally, improved communication skills, education of health professionals, and patient education are the most important PE facilitators [30,31]. In line with this, the results from a previous mixed methods study indicated that an emphasis on the post-discharge rehabilitation process significantly relied not only on the behaviors and the communication skills of individual providers but also on the support from team leaders as well as the local institutional context [28]. Consequently, forthcoming initiatives aimed at enhancing patient involvement in the rehabilitation process should concentrate on the meso-level. This involves implementing training programs for health professionals in patient–professional cooperation, refining referral routines, enhancing information flow among providers and affiliated services, and ensuring adequate competence and human resources at every level of care.

Our results demonstrated that the vast majority of the patients included were actively involved in setting goals and preparing their rehabilitation plans. However, the findings also showed a reduced likelihood of men and older individuals actively participating in developing rehabilitation goals. Results from a recent national survey demonstrated that health literacy declines with advancing age [32]. One factor contributing to the lower engagement among older individuals may therefore be lower health literacy, which impacts their capability and desire to actively engage in the rehabilitation process [33]. Other factors may include low resilience, reduced intrinsic capacity, and depressed moods among older persons [34,35]. Consequently, future goal-setting initiatives should recognize the significance of promoting mental well-being in addition to physical function [36]. Another explanation may be ageism, meaning that health professionals may expect less involvement or engage elderly people in setting goals to a lesser extent. Additionally, it was previously common practice that multidisciplinary teams would formulate goals on behalf of patients; therefore, based on experience, older individuals may expect professionals to set achievable goals on their behalf. However, this paternalistic patient–therapist dynamic contradicts current national strategies that underscore the importance of involving patients in their rehabilitation process [37]. Addressing the trend toward diminished engagement among men and older patients necessitates a proactive meso-level approach. This can be achieved, for instance, by implementing methods specifically designed to encourage patients to take a more active approach [38,39].

An important finding in our study is that function, as measured through the PSFS, improved in both groups over the one-year follow-up period. Interestingly, participants who actively engaged in goalsetting exhibited a more substantial enhancement in function, which is in line with findings from a recent study [40]. Additionally, these participants had significantly greater PSFS scores from baseline to the 12-month post-rehabilitation assessment. One reason for this finding may be that rehabilitation goals that individuals have ownership of motivate them to exert a high level of personal effort to achieve these goals both during and after the inpatient rehabilitation period. Another explanation may be the focus on activity and participation-related goals enhanced through the use of the PSFS as such goals have been found to be associated with better outcomes [41].

We also found that involvement in goal-setting almost tripled the odds of goal attainment and that being involved in developing their rehabilitation plan nearly doubled the odds of attaining their own rehabilitation goals. These findings are comparable with the results of a previous study demonstrating a positive association between PE and goal attainment after rehabilitation [42]. Taken together, the results from our study point to the importance of facilitating patient involvement as engaged patients benefit more from rehabilitation than those who do not.

A strength of this study is the large sample recruited from a large number of rehabilitation centers across Norway. The results may therefore be relevant to people with a wide variety of diseases and to rehabilitation in various contexts. However, as participants were recruited from rehabilitation institutions in Norway, the results may not necessarily be generalizable to other countries or healthcare systems.

Another strength is the use of patient-reported outcome measures as these are believed to complement physician-rated outcomes in the sense of coming closer to patients’ preferred levels of involvement [43,44]. It is essential to acknowledge certain limitations that may impact the generalizability and interpretability of the findings. The data included were limited to the population referred to specialized rehabilitation, which may be a different population with different functional problems than patients who receive rehabilitation in hospitals or primary care. Patients in specialized rehabilitation may be prone to having more stable conditions and higher functional levels than those receiving rehabilitation in hospitals or primary care. Second, people with poor oral or written Norwegian language skills were not included. Third, the study results are affected by the outcome measures used. The reliance on patient-reported outcomes in this study may have introduced constraints in capturing a comprehensive understanding of patient function and rehabilitation outcomes. Self-reported data are at risk of recall bias and response bias, such as response shift, and the natural tendency for people to overstate social acts, such as the degree of goal attainment and/or involvement during the rehabilitation process [45,46,47]

The majority of participants actively engaged in their rehabilitation process as well as those who participated in goal-setting and planning experienced more favorable outcomes compared with those who did not. To improve patient involvement in rehabilitation, future initiatives should focus on the meso-level. This involves implementing training programs that enhance the communication skills of healthcare professionals, ensuring effective information flow among providers and affiliated services, and ensuring sufficient competence and human resources at every level of care.

## Figures and Tables

**Table 1:** Baseline characteristics of all participants (*n* = 2113).

Women, *n*, %	1470	70
Age, years, mean, SD	53.5	13.5
Living with partner, *n*, %	1080	58
Higher education ≥ 12 years, *n*, %	1035	49
Full-time paid work, *n*, %	620	29
Part-time paid work, *n*, %	432	20
Not in paid work, *n*, %	789	43
Pain ≥ 3 months, *n*, %	1418	92
RMDs *, *n*, %	1117	53
Lifestyle disease, *n*, %	235	11
Neurological injuries and diseases, *n*, %	270	10
Other diseases or injuries, *n*, %	491	23

* RMD: rheumatic and musculoskeletal diseases. Frequencies, percentages, mean, and standard deviation (SD).

**Table 2:** Patient characteristics and involvement in the rehabilitation process (total *n* = 2113) are reported as proportions.

	Actively Involved in Goal-Setting *n* = 2007/95%	Actively Involved in Preparing the Rehab Plan *n* = 1666/79%	Participated in at Least Two Meetings *n* = 774/84%	Relatives Invited to Participate in Meetings *n* = 478/23%	External Professionals Invited to Participate in Meetings *n* = 391/19%
	%	%	%	%	%
Male	92	77	84	27	22
Female	96	80	84	21	17
≥18–39 yrs.	96	84	93	34	26
40–49 yrs.	97	84	87	26	25
50–59 yrs.	96	84	87	22	19
60–69 yrs.	95	75	81	18	11
70–>80 yrs.	89	61	68	10	6
Single	95	76	84	19	18
Living with partner	95	81	84	24	18
Higher education ≥ 12 Years	95	79	86	22	19
Education ˂ 12 years	96	80	82	22	16
Full-time paid work	96	81	87	20	16
Part-time paid work	96	83	84	26	23
Not in paid work	93	76	82	21	17
Pain ˂ 3 months	92	71	73	22	13
Pain ≥ 3 months	95	79	86	21	19
RMDs *	95	78	83	18	18
Lifestyle disease	95	83	88	24	14
Neurological injuries and diseases	94	75	82	30	19
Other diseases or injuries	95	82	86	29	22

* RMDs: Rheumatic and musculoskeletal diseases. Frequencies and percentages.

**Table 3:** Associations between patient characteristics and involvement in goal-setting (yes/no) (*n* = 1531).

		95% CI for OR		
	OR	Lower	Upper	*p*-Value
Sex, women ^ref men^	1.87	1.15	3.02	0.01
Age	0.97	0.95	0.99	0.004
Living with partner ^ref single^	0.95	0.59	1.53	0.85
Higher education ≥ 12 years ^ref˂12 months^	0.77	0.47	1.24	0.28
Part-time paid work ^ref not in work^	1.71	0.89	3.27	0.10
Full-time paid work ^ref not in work^	1.70	0.96	2.99	0.066
˂3 months of pain ref ^≥3 months pain^	0.66	0.32	1.35	0.26
RMDs * ^ref other^	1.23	0.69	2.20	0.47
Lifestyle disease ^ref other^	1.00	0.41	2.46	0.99
Neurological diseases or injuries ^ref other^	1.11	0.48	2.57	0.79

* RMDs: Rheumatic and Musculoskeletal Diseases. Logistic regression.

**Table 4:** Associations between patient characteristics, involvement in the rehabilitation process, and goal attainment (*n* = 1501).

		95% CI for OR		
	OR	Lower	Upper	*p*-Value
Sex, women ^ref men^	1.26	0.92	1.76	0.14
Age	1.03	1.02	1.04	<0.001
Living with partner ^ref single^	1.06	0.81	1.40	0.64
Higher education ≥ 12 years ^ref ˂ 12 months^	0.71	0.54	0.95	0.02
Work participation				
Part-time paid work ^ref not in paid work^	1.20	0.85	1.69	0.28
Full-time paid work ^ref not in paid work^	1.34	0.97	1.85	0.74
˂3 months of pain ^ref ≥3 months of pain^	2.17	1.17	4.00	0.01
RMDs * ^ref other^	1.09	0.76	1.56	0.63
Neurological diseases ^ref other^	0.79	0.49	1.28	0.34
Lifestyle disease ^ref other^	4.93	2.35	10.35	<0.001
Involvement in the rehabilitation process				
Actively involved in goal-setting	2.79	1.60	4.87	<0.001
Actively involved in the rehab plan	2.04	1.44	2.87	<0.001
Participated in a minimum of two meetings	1.25	0.84	1.84	0.25
Next of kin asked to participate in meetings	1.22	0.84	1.77	0.29
External personnel asked to participate in meetings	1.08	0.74	1.57	0.68

* RMDs: Rheumatic and musculoskeletal disease. Logistic regression.

## Data Availability

The dataset is available from the corresponding author upon reasonable request.
